# Early exposure to hyperoxia and mortality in critically ill patients with severe traumatic injuries

**DOI:** 10.1186/s12890-017-0370-1

**Published:** 2017-02-03

**Authors:** Derek W. Russell, David R. Janz, William L. Emerson, Addison K. May, Gordon R. Bernard, Zhiguo Zhao, Tatsuki Koyama, Lorraine B. Ware

**Affiliations:** 1 Lung Health Center, Division of Pulmonary and Critical Care Medicine, University of Alabama at Birmingham, 1900 University Blvd., THT 423, Birmingham, AL 35233 USA; 2 Section of Pulmonary and Critical Care Medicine, Louisiana State University School of Medicine New Orleans, New Orleans, LA USA; 3 Departments of Medicine and Pathology, Microbiology and Immunology, Vanderbilt University, Nashville, TN USA

## Abstract

**Background:**

Hyperoxia is common early in the course of resuscitation of critically ill patients. It has been associated with mortality in some, but not all, studies of cardiac arrest patients and other critically ill cohorts. Reasons for the inconsistency are unclear and may depend on unmeasured patient confounders, the timing and duration of hyperoxia, population characteristics, or the way that hyperoxia is defined and measured. We sought to determine whether, in a prospectively collected cohort of mechanically ventilated patients with traumatic injuries with and without head trauma, higher maximum partial pressure of arterial oxygen (PaO2) within 24 hours of admission would be associated with increased risk of in-hospital mortality.

**Methods:**

Critically ill patients with traumatic injuries undergoing invasive mechanical ventilation enrolled in the Validating Acute Lung Injury biomarkers for Diagnosis (VALID) study were included in this study. All arterial blood gases (ABGs) from the first 24 hours of admission were recorded. Primary analysis was comparison of the highest PaO2 between hospital survivors and non-survivors.

**Results:**

A total of 653 patients were evaluated for inclusion. Of these, 182 were not mechanically ventilated or did not have an ABG measured in the first 24 hours, leaving 471 patients in the primary analysis. In survivors, the maximum PaO2 was 141 mmHg (median, interquartile range 103 - 212) compared to 148 mmHg (IQR 105 - 209) in non-survivors (*p* = 0.82). In the subgroup with head trauma (n = 266), the maximum PaO2 was 133 mmHg (IQR 97 - 187) among survivors and 152 mmHg (108 - 229) among nonsurvivors (*p* = 0.19). After controlling for age, injury severity score, number of arterial blood gases, and fraction of inspired oxygen, maximum PaO2 was not associated with increased mortality (OR 1.27 for every fold increase of PaO2 (95% CI 0.72 - 2.25).

**Conclusions:**

In mechanically ventilated patients with severe traumatic injuries, hyperoxia in the first 24 hours of admission was not associated with increased risk of death or worsened neurological outcomes in a setting without brain tissue oxygenation monitoring.

**Electronic supplementary material:**

The online version of this article (doi:10.1186/s12890-017-0370-1) contains supplementary material, which is available to authorized users.

## Background

Trauma accounts for approximately 10% of deaths worldwide and causes a disproportionately high amount of disability and lost years of life [1]. The magnitude of this problem may be even greater than recognized, as a significant number of patients survive hospitalization but ultimately succumb to complications of their injuries [2]. Therefore, improvements in management of patients with severe traumatic injury are needed.

Exposure to supraphysiologic levels of partial pressure of arterial oxygen (hereafter “hyperoxia”) is common in mechanically ventilated, critically ill patients [3–9]. The effect of hyperoxia on outcomes in a non-cardiac arrest population of critically ill patients remains unknown. Patients with severe traumatic injury may be especially susceptible to detrimental neurological effects of hyperoxia due to the prevalence of traumatic brain injury (TBI) in this population, but the biological and clinical effects of hyperoxia are complex and incompletely understood [3–5, 10–12]. The observed prevalence and degree of hyperoxia vary widely among centers [3–9] and have been associated with both better [13, 14] and worse [3, 6–9] outcomes in critically ill adults.

The association of hyperoxia with outcome has been previously studied in patients with critical traumatic injury. One large retrospective cohort analysis of TBI patients showed a U-shaped relationship between in-hospital and 6 month mortality and arterial oxygen tension in the first 48 h of admission, but there was no significant relationship between hyperoxia and mortality on multivariate analysis [15]. In this study, analysis was limited to arterial blood gases recorded according to APACHE II methodology (i.e., the highest alveolar-arterial gradient for patients on FiO_2_ 0.5 or greater, or the lowest PaO_2_ for patients on FiO_2_ < 0.5). This approach would tend to select for the blood gas associated with lowest oxygen tension and thus may not be ideal for measurement of any potential effects of early hyperoxia on outcomes. In another analysis focusing on PaO_2_ in the first 24 h, there was an independent association between mortality and hyperoxia (defined as PaO_2_ > 300 mmHg) in a TBI cohort [9]. Thus, data is mixed on this question perhaps due to differences in the time point analyzed and approach to assessing arterial oxygen tension.

Because the peri-contusional brain tissue in TBI is at risk for hypoxic-ischemic injury, strategies have been developed to directly measure the partial pressure of oxygen in brain tissue (PBrO_2_) using brain probe monitors to guide oxygen management via adjustments in FiO_2_ and/or mean airway pressure. A small phase II RCT suggests improved outcomes with such an approach [16] and results of a larger RCT studying an aggressive PBrO_2_-guided oxygen management are expected soon [17]. However, these techniques are not yet universally adopted and may not be possible in resource-limited settings. Furthermore, even at institutions using this modality, there is an early period of resuscitation that occurs prior to implantation of PBrO_2_ monitors. Consequently, the safe range of PaO_2_ in critically ill adults without brain tissue oxygenation monitoring early after traumatic injury is unclear.

We performed a retrospective cohort study in mechanically ventilated patients with severe traumatic injuries to test the hypothesis that the maximum measured partial pressure of arterial oxygen (PaO_2_) within 24 h of admission would be associated with an increased risk of in-hospital mortality and that this association would be stronger in patients with head injury.

## Methods

### Patients

**Fig. 1:**
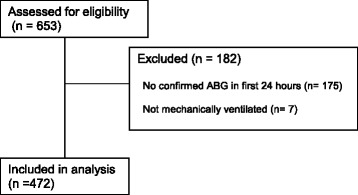
CONSORT Diagram. Of 653 patients admitted to the Trauma ICU during the enrollment period and assessed for eligibility, 471 had an ABG in the first 24 h from admission and were mechanically ventilated; these patients were included in the analysis

The study population consisted of 653 consecutive patients who were prospectively enrolled in the Validating Acute Lung Injury Markers for Diagnosis (VALID) study on the morning after admission to the Trauma ICU. Methods for the VALID study have been previously published [18–20], and the Vanderbilt Institutional Review Board approved the study. The VALID study included critically ill patients who were ≥18 years old and who were admitted to Vanderbilt ICUs for at least 2 days. Of the 653 patients, 182 were excluded from the analysis as they were not receiving invasive mechanical ventilation or did not have a confirmed arterial blood gas (ABG) measured in the first 24 h of admission (Fig. 1).

### Measurements

All arterial blood gas analysis measurements obtained through arteriopuncture or arterial catheters for clinical purposes during the first 24 h of admission were recorded. The maximum PaO_2_ in the 24 h after admission, the corresponding fraction of inspired oxygen (FiO_2_), and the number of ABGs measured during the 24 h after admission were also recorded. Other data collection included the APACHE II score [21], the injury severity score (ISS) [22] and the presence or absence of head injury (i.e., any traumatic insult to the face, neck, cranium, or intracranial contents). Head injury was noted to be present or absent based on the report or electronic medical record documentation of the bedside attending physician. The Glasgow Coma Scale (GCS) score [23] at time of hospital discharge was also included for analysis, as this has been shown to act as a reasonable surrogate for long-term neurologic outcomes in patients with traumatic brain injury [24].

### Statistical analysis

Patients’ baseline characteristics were summarized by median with interquartile range for continuous variables and frequencies with percentages for categorical variables. To compare patient’s characteristics between in-hospital mortality groups (survivors vs. non-survivors), Wilcoxon rank-sum test (continuous variables) and Pearson *χ* ^2^ test (categorical variables) were used. In the primary analysis, association between the highest PaO_2_ measured in the 24 h after admission and in-hospital mortality was evaluated using a logistic regression model controlling for age, injury severity score, number of measured ABGs, and fraction of inspired oxygen. The ISS and age were included in the model because they are known to have associations with outcome in this population. The number of measured ABGs was included because the timing and number of ABG assessments was not standardized in this study and is an inherent confounder of the likelihood of having a high PaO_2_ measurement. As clinicians may be more likely to check an ABG (and therefore more likely to document hyperoxia) in patients who are receiving a high FiO_2_, this was also adjusted for in the analysis. GCS was not included in the adjustment model because this measure is heavily affected by both sedation practices and TBI and thus may not be internally consistent in its interaction with outcomes of interest in this population. As a secondary investigation, the association between the highest PaO_2_ and the Glasgow Coma Scale (GCS) at time of discharge was evaluated using a proportional odds model adjusting for the same set of factors as in the primary analysis. A planned subgroup analysis of those with head injury was also conducted and reported.

In all models, the maximum PaO_2_ values were transformed using logarithmic function. Missing baseline values were imputed using a multiple imputation method (30-iteration) [25]. Statistical analysis was conducted with R version 3 (R Core Team) [26]; a two-sided significance level of 0.05 was used for statistical inference.

## Results

### Clinical characteristics

The patient population consisted exclusively of critically ill patients with severe traumatic injuries admitted to the Vanderbilt Trauma ICU consecutively from February 2006 to February 2011 (Table 1). summarizes characteristics of survivors and non-survivors. Survivors were significantly younger, had higher Glasgow Coma Scale (GCS) scores and lower APACHE II scores on admission. Gender, ISS, number of ABGs measured, FiO_2_, and maximum PaO_2_ were not significantly different between the two groups.

**Table 1:** Baseline characteristics of 471 critically-Ill trauma patients included in the analysis

Characteristic	*N*	Overall	Survivors	Non-survivors	*p*-value
		*n* = 471	*n* = 422 (89.6%)	*n* = 49 (10.4%)	
Age (Years)	471	42 (27, 55)	41 (27, 54)	51 (43, 67)	<0.001
Men (n, %)	471	342 (73%)	308 (73%)	34 (69%)	0.59
Head Trauma (Yes)	471	266 (56%)	231 (55%)	35 (71%)	0.03
APACHE II score	471	25 (20, 28)	24 (20, 28)	28 (25, 31)	<0.001
Injury Severity Score	470	29 (18, 36)	29 (18, 36)	29 (20, 39)	0.37
Number of ABGs measured	471	3 (2, 5)	3 (2, 5)	3 (2, 6)	0.44
FiO_2_ Associated with Maximum PaO_2_	352	0.40 (0.40, 0.60)	0.40 (0.40, 0.60)	0.40 (0.40, 0.60)	0.56
Maximum PaO_2_ (mmHg)	471	142 (103, 212)	141 (103, 212)	148 (105, 209)	0.82
GCS	471	11 (8, 15)	11 (9, 15)	3 (3, 9)	<0.001

### In-hospital mortality and maximum PaO_2_

**Fig. 2:**
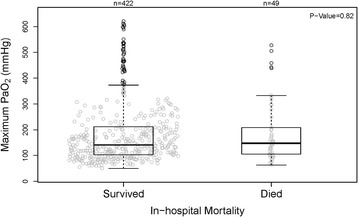
No association was seen between maximum PaO_2_ in the first 24 h after admission and in hospital mortality in an unadjusted analysis

Among the 471 patients in the primary analysis, 49 (10.4%) died during hospitalization. There was no difference between the maximum PaO_2_ of survivors [median 141 mmHg (IQR 103–212)] and nonsurvivors [148 (105–209), *p* = 0.82] (Fig. 2). A logistic regression model adjusting for potential confounders showed no association of maximum PaO_2_ with mortality, with an adjusted odds ratio for every fold increase of PaO_2_ of 1.27 (95% CI 0.72–2.25) (Table 2).

**Table 2:** Logistic regression model for in-hospital mortality

Characteristic	Odds ratio	95% Confidence interval	*p*-value
Age (Increment of 5 years)	1.20	1.10–1.31	<0.001
Injury Severity Score (Increment of 5)	1.41	1.03–1.94	0.03
Number of ABGs Measured	1.05	0.91–1.22	0.49
FiO_2_ at time of ABG (Increment of 10%)	0.94	0.77–1.15	0.54
Maximum PaO_2_ (Increment of 1 fold)	1.27	0.72–2.25	0.41

### Head injury subgroup

As the effect of hyperoxia might differ between patients with head injury and those without, we conducted a subgroup analysis to determine if the adjusted association between mortality and maximum PaO_2_ was different for those with head injury. Of the entire cohort of 471 patients analyzed for this study, 266 (56.5%) carried a diagnosis of head injury. In this subgroup, the maximum PaO_2_ among survivors was 133 mmHg (97–187) compared to 152 mmHg (108–229) among nonsurvivors (*p* = 0.19). After controlling for age, ISS, number of ABGs measured, and the FiO_2_ at the time of PaO_2_measurement, there was no significant association between maximum PaO_2_ and mortality in patients with or without head trauma (OR 1.55, 95% CI 0.79–3.02, *p* = 0.20, and OR 1.01, 95% CI 0.31–3.28 *p* = 0.98, respectively) (Table 3). Head injury did not modify the effect of PaO_2_ on mortality (*p* = 0.21).

**Table 3:** Logistic regression model for in-hospital mortality in head trauma patients

Characteristic	Odds ratio	95% Confidence interval	*p*-value
Age (Increment of 5 years)	1.22	1.09–1.36	<0.001
Injury Severity Score (Increment of 5)	1.28	0.86–1.91	0.23
Number of ABGs Measured	1.15	0.98–1.37	0.10
FiO_2_ at time of ABG (Increment of 10%)	0.88	0.69–1.12	0.29
Maximum PaO_2_ (Increment of 1 fold)	1.55	0.79–3.02	0.20

To study the potential association between PaO_2_ and neurologic outcomes, Glasgow Coma Scale (GCS) scores were collected on the day of hospital discharge for all patients. After adjusting for potential confounders, there was no association between the maximum PaO_2_ measured within 24 h of admission and the GCS measured on the day of hospital discharge (OR for maximum PaO_2_ 0.96, 95% CI 0.71–1.31, *p* = 0.82). When this analysis was restricted to patients with and without head injury, there was still no association between maximum PaO_2_ and lower GCS at discharge (OR 1.10, 95% CI 0.76–1.60 *p* = 0.62 in the head injury group and OR 0.66, 95% CI 0.38–1.15 *p* = 0.14 in the non head injury group [Additional file 1: Tables S1, Additional file 2: Tables S2 and Additional file 3: Tables S3]. The presence of head injury did not modify the effect of PaO_2_ on GCS at hospital discharge (*p* = 0.11) (Table 4).

**Table 4:** Proportional odds regression model for GCS

Characteristic	Odds ratio^a^	95% Confidence interval	*p*-value
Age (Increment of 5 years)	1.05	1.00–1.10	0.04
Injury Severity Score (Increment of 5)	1.24	1.05–1.48	0.01
Number of ABGs Measured	1.11	1.02–1.21	0.01
FiO_2_ at time of ABG (Increment of 10%)	0.93	0.83–1.05	0.25
Maximum PaO_2_ (Increment of 1 fold)	0.96	0.71–1.31	0.82

^a^odds ratio for lower GCS

APACHE II score was not included in the reported logistic regression models because PaO_2_ is a component of APACHE II_._ However, since APACHE II score was associated with mortality on univariate analysis, a sensitivity analysis that included APACHE II as a variable was done for all models. Inclusion of APACHE II did not alter the reported conclusions; these analyses are reported in [Additional file 4: Table S4, Additional file 5: Table S5, Additional file 6: Table S6].

## Discussion

Contrary to our hypothesis, we did not find an association between increased PaO_2_ within 24 h of ICU admission and in-hospital mortality in mechanically ventilated patients with severe traumatic injuries. Additionally, we did not detect such an association in the subgroup of patients with head injury. These findings provide reassuring evidence that hyperoxia early in the course of severe traumatic injury does not have major adverse effects.

It is worth noting that arterial hyperoxia as measured in this study is distinct from cerebral hyperoxia (i.e., elevated oxygen tension in brain tissue). PBrO_2_ monitoring in individuals with TBI allows for an individualized approach to oxygen targets in TBI patients [27–29] and its use may obviate concern for brain tissue oxygen toxicity in this population. However, this technology is not available at all centers. Furthermore, in the early period preceding insertion of the PBrO_2_ monitor, the management of oxygenation is still empiric. It is tempting to assume that a liberal oxygen target is best in these patients because of the high incidence of cerebral hypo-oxygenation, as is recommended in pre-hospital trauma care guidelines [29]. However, there may be some patients who would not benefit or would be harmed by such an approach; therefore, this question required further study. Our analysis affirms a prior report that such an approach is indeed safe [15]. However, because the literature is mixed as to the effects of hyperoxia in TBI and our study was not powered to exclude a small effect in this secondary subgroup analysis, further investigation remains warranted.

The significance of these findings is most apparent when considered in light of the available literature on this topic. Considerable evidence has accrued that hyperoxia can have harmful biochemical and physiological effects. Elevated PaO_2_ may increase the formation of reactive oxygen species (ROS) in the neuronal tissue bed, favoring the induction of neuronal cell death, and potentially contributing to poor neurological outcomes [10, 11]. Supraphysiologic levels of PaO_2_ can also cause cerebral vasoconstriction and heterogeneous tissue bed perfusion patterns, potentially resulting in paradoxically lower delivery of oxygen and other important substrates to cerebral tissue [11, 30, 31].

Conversely, a beneficial effect of increased PaO_2_ has been postulated for certain patient groups [14, 32]. Human studies have associated hyperoxia with improvements in intracranial pressure, tissue bed oxygenation in both peri-contusional and remote neuronal tissue, and more aerobic neural metabolic profiles in patients with TBI [14]. Early reductions in neurologic deficits and radiographic patterns of injury in patients with stroke who were exposed to hyperoxia have also been reported, supporting a potentially neuroprotective role for hyperoxia in the injured brain [32].

In previous cohort studies, hyperoxia has been associated with increased mortality in a variety of clinical settings [3, 5–9, 33] but these associations have not been seen in all studies [4, 15, 34–37]. Data in the general mechanically ventilated population have been mixed with the largest retrospective cohort study to date finding no effect [4, 5]. A recent meta-analysis found arterial hyperoxia to correlate with mortality but noted substantial heterogeneity of effect that could be due to differences in study design [38].

In light of this heterogeneity, several aspects of the current study enhance the validity of its findings. One strength of this analysis is that all ABGs obtained in the first 24 h after admission were analyzed rather than just those that contributed to APACHE scoring. Furthermore, PaO_2_ was analyzed as a continuous variable with the odds ratio of mortality for every fold increase of PaO_2_ above 50 mmHg reported. This measurement of oxygen exposure differs from that used in other studies, and may address some of the limitations of prior reports. Past studies of hyperoxia have used an arbitrary PaO_2_ cutoff of 200 or 300 mmHg, quintiles of hyperoxia exposure, or in the minority, PaO2 as a continuous variable [3–7, 9]. We chose to analyze PaO_2_ as a continuous variable because hyperoxia has no consensus definition, arbitrary partial pressure cut-off values may not accurately reflect biology, and because it increases statistical power. Analysis of the data using an arbitrary cutoff that has been used in some prior studies (300 mmHg) did not change our results (data not shown). The highest PaO_2_ during the first 24 h was chosen as a marker of exposure to hyperoxia for this trial. This early time point was chosen because hyperoxia is more common in our center during the early period. Also, vulnerable tissues may be most susceptible to any ill effects of hyperoxia early after trauma. Other publications have examined a varied range of time points and, in some cases, only analyzed the ABG with the lowest alveolar-arterial gradient, lowest PaO_2,_ or average PaO_2,_ thus potentially missing many cases of hyperoxia exposure [8, 15]. This early time point also increases the relevance of our results to the empiric management of oxygen prior to brain tissue monitor placement in centers with PbrO_2_ monitoring capability.

Our study is subject to some limitations. The retrospective design limits interpretation of the results. Although about half of our patients were classified as having head injury, this diagnosis was defined as the bedside physician noting head injury in the medical record and likely includes patients with varying degrees of trauma to the neck, face, and cranium. Sufficient data were not collected as part of the VALID study to know whether patients had true TBI. Other explanations for not finding a significant difference in PaO_2_ between survivors and non-survivors include a relatively low median PaO_2_ observed (142 mmHg) in our entire cohort compared with past studies of oxygen exposure (major studies ranging from 99 to 247 mmHg) [3–9]. Additionally, it is not known if risk accumulates over time with continuing exposure to hyperoxia, and our study was not designed to assess for such an effect. Furthermore, our study may have been underpowered to detect a small effect of hyperoxia. With our sample size of 471, we would have had 80% power to detect an association if the odds ratio corresponding to one-standard-deviation increase in maximum PaO_2_ were 1.53. To achieve 80% power to detect our observed OR of 1.27 would require *N* = 1973. Finally, there may be outcomes associated with increased PaO_2_ other than mortality that the current study did not analyze, including neurologic outcomes, which have previously been shown to worsen in association with exposure to hyperoxia [6]. However, GCS at time of discharge has been shown to be a reasonable surrogate for longer-term neurologic outcomes [24] and a lower GCS did not associate with maximum PaO_2_ in our cohort.

## Conclusions

The optimal oxygenation strategy in critically ill patients with traumatic injuries who do not have brain tissue monitoring remains unknown. This study finds no association of hyperoxia with mortality or lower GCS at discharge in a population of mechanically ventilated patients who are critically ill with traumatic injuries. Our findings are reassuring that liberal oxygen supplementation can be safely applied in the management of the general trauma ICU population as well as in patients with head injury. However, these findings bear confirmation in a larger cohort of TBI patients, because our study was underpowered to exclude a small effect in this subgroup. When viewed in light of previous research in this area showing harm from hyperoxia in some populations and not others, this study supports the hypothesis that the association of higher PaO_2_ with mortality depends on the nature of the patient population studied.

## Additional files

Additional file 1: Table S1. Logistic regression model for in-hospital mortality in patients without head injury. (DOCX 14 kb)
Additional file 2: Table S2. Proportional odds regression model for GCS at discharge in patients with head injury. (DOCX 14 kb)
Additional file 3: Table S3. Proportional odds regression model for GCS at discharge in patients without head injury. (DOCX 14 kb)
Additional file 4: Table S4. Logistic regression model for in-hospital mortality (including APACHE). (DOCX 14 kb)
Additional file 5: Table S5. Logistic regression model for in-hospital mortality in patients with head injury (including APACHE). (DOCX 14 kb)
Additional file 6: Table S6. Proportional odds regression model for GCS at discharge (including APACHE). (DOCX 14 kb)
