# Supplementary material for: Early exposure to hyperoxia and mortality in critically ill patients with severe traumatic injuries
Source: BMC Pulm Med. 2017 Feb 3;17:29. doi: 10.1186/s12890-017-0370-1 (PMC5291954; doi:10.1186/s12890-017-0370-1)
Supplement: Additional file 1: Table S1. — Logistic regression model for in-hospital mortality in patients without head injury. (DOCX 14 kb) [file 12890_2017_370_MOESM1_ESM.docx]

**Additional Files**

| **Additional File 1: Table 1S. Logistic Regression Model for In-Hospital Mortality in Patients without Head Injury** | | | |  |
| --- | --- | --- | --- | --- |
| **Characteristic** | **Odds Ratio** | **95% Confidence Interval** | ***p-value*** | |
| Age (Increment of 5 years) | 1.21 | 1.03-1.41 | 0.02 | |
| Injury Severity Score (Increment of 5) | 1.33 | 0.74-2.39 | 0.34 | |
| Number of ABGs Measured | 0.943 | 0.68-1.29 | 0.69 | |
| FiO_2_ at time of ABG (Increment of 10%) | 1.02 | 0.68-1.54 | 0.92 | |
| Maximum PaO_2_ (Increment of 1 fold) | 1.01* | 0.31-3.28 | 0.98 | |
|  |  |  |  | |
|  |  |  |  | |

*The reported odds ratio for Maximum PaO2 is for every fold increase in PaO_2_ above 50mmHg.
