# Supplementary material for: Early exposure to hyperoxia and mortality in critically ill patients with severe traumatic injuries
Source: BMC Pulm Med. 2017 Feb 3;17:29. doi: 10.1186/s12890-017-0370-1 (PMC5291954; doi:10.1186/s12890-017-0370-1)
Supplement: Additional file 2: Table S2. — Proportional odds regression model for GCS at discharge in patients with head injury. (DOCX 14 kb) [file 12890_2017_370_MOESM2_ESM.docx]

| **Table**  **Table Additional File 2: Table 2S. Proportional Odds Regression Model for GCS in Patients with Head Injury** | | |  | |  |
| --- | --- | --- | --- | --- | --- |
| **Characteristic** | **Odds Ratio^1^** | **95% Confidence Interval** | | ***p-value*** | |
| Age (Increment of 5 years) | 1.03 | 0.97-1.10 | | 0.33 | |
| Injury Severity Score (Increment of 5) | 1.26 | 1.00-1.60 | | 0.05 | |
| Number of ABGs Measured | 1.07 | 0.95-1.20 | | 0.25 | |
| FiO_2_ at time of ABG (Increment of 10%) | 0.89 | 0.77-1.03 | | 0.13 | |
| Maximum PaO_2_ (Increment of 1 fold) | 1.10 | 0.76-1.60 | | 0.62 | |
|  |  |  | |  | |
|  |  |  | |  | |
| ^1^ Odds ratio for lower GCS. | | |  | |  |
