# Supplementary material for: Early exposure to hyperoxia and mortality in critically ill patients with severe traumatic injuries
Source: BMC Pulm Med. 2017 Feb 3;17:29. doi: 10.1186/s12890-017-0370-1 (PMC5291954; doi:10.1186/s12890-017-0370-1)
Supplement: Additional file 3: Table S3. — Proportional odds regression model for GCS at discharge in patients without head injury. (DOCX 14 kb) [file 12890_2017_370_MOESM3_ESM.docx]

| **Additional File 3: Table 3S. Proportional Odds Regression Model for GCS in Patients without Head Injury** | | |  |
| --- | --- | --- | --- |
| **Characteristic** | **Odds Ratio^1^** | **95% Confidence Interval** | ***p-value*** |
| Age (Increment of 5 years) | 1.14 | 1.06-1.23 | <0.001 |
| Injury Severity Score (Increment of 5) | 1.09 | 0.82-1.45 | 0.55 |
| Number of ABGs Measured | 1.35 | 1.18-1.55 | <0.001 |
| FiO_2_ at time of ABG (Increment of 10%) | 1.04 | 0.86-1.25 | 0.69 |
| Maximum PaO_2_ (Increment of 1 fold) | 0.66 | 0.38-1.15 | 0.14 |
|  |  |  |  |
|  |  |  |  |
| ^1^ Odds ratio for lower GCS. | | |  |
