# Supplementary material for: Early exposure to hyperoxia and mortality in critically ill patients with severe traumatic injuries
Source: BMC Pulm Med. 2017 Feb 3;17:29. doi: 10.1186/s12890-017-0370-1 (PMC5291954; doi:10.1186/s12890-017-0370-1)
Supplement: Additional file 4: Table S4. — Logistic regression model for in-hospital mortality (including APACHE). (DOCX 14 kb) [file 12890_2017_370_MOESM4_ESM.docx]

| **Additional File 4: Table 4S. Logistic Regression Model for In-Hospital Mortality (including APACHE)** | | |  |
| --- | --- | --- | --- |
| **Characteristic** | **Odds Ratio** | **95% Confidence Interval** | ***p-value*** |
| Age (Increment of 5 years) | 1.15 | 1.05-1.25 | 0.002 |
| Injury Severity Score (Increment of 15) | 1.31 | 0.94-1.82 | 0.11 |
| Number of ABGs Measured | 0.97 | 0.83-1.13 | 0.67 |
| FiO_2_ at time of ABG (Increment of 10%) | 0.90 | 0.72-1.12 | 0.34 |
| Maximum PaO_2_ (Increment of 1 fold ) | 1.36 | 0.75-2.46 | 0.31 |
| APACHE at enrollment (Increment of 5) | 1.68 | 1.26-2.26 | <0.001 |
|  |  |  |  |
|  | | |  |
