# Supplementary material for: Early exposure to hyperoxia and mortality in critically ill patients with severe traumatic injuries
Source: BMC Pulm Med. 2017 Feb 3;17:29. doi: 10.1186/s12890-017-0370-1 (PMC5291954; doi:10.1186/s12890-017-0370-1)
Supplement: Additional file 5: Table S5. — Logistic regression model for in-hospital mortality in patients with head injury (including APACHE). (DOCX 14 kb) [file 12890_2017_370_MOESM5_ESM.docx]

| **Additional File 5: Table 5S. Logistic Regression Model for In-Hospital Mortality in Head Trauma Patients (including APACHE)** | | | |
| --- | --- | --- | --- |
| **Characteristic** | **Odds Ratio** | **95% Confidence Interval** | ***p-value*** |
| Age (Increment of 5 years) | 1.17 | 1.04-1.31 | 0.007 |
| Injury Severity Score (Increment of 5) | 1.15 | 0.75-1.75 | 0.52 |
| Number of ABGs Measured | 1.09 | 0.91-1.30 | 0.34 |
| FiO_2_ at time of ABG (Increment of 10%) | 0.85 | 0.66-1.10 | 0.22 |
| Maximum PaO_2_ (Increment of 1 fold) | 1.56 | 0.78-3.11 | 0.21 |
| APACHE at enrollment (Increment of 5) | 1.58 | 1.10-2.27 | 0.01 |
|  |  |  |  |
|  | | |  |
