# Supplementary material for: Early exposure to hyperoxia and mortality in critically ill patients with severe traumatic injuries
Source: BMC Pulm Med. 2017 Feb 3;17:29. doi: 10.1186/s12890-017-0370-1 (PMC5291954; doi:10.1186/s12890-017-0370-1)
Supplement: Additional file 6: Table S6. — Proportional odds regression model for GCS at discharge (including APACHE). (DOCX 14 kb) [file 12890_2017_370_MOESM6_ESM.docx]

| **Table Additional File 6: Table 6S. Proportional Odds Regression Model for GCS (including APACHE)** | | |  | |  |
| --- | --- | --- | --- | --- | --- |
| **Characteristic** | **Odds Ratio^1^** | **95% Confidence Interval** | | ***p-value*** | |
| Age (Increment of 5 years) | 1.03 | 0.98-1.07 | | 0.29 | |
| Injury Severity Score (Increment of 5) | 1.18 | 0.99-1.41 | | 0.06 | |
| Number of ABGs Measured | 1.06 | 0.97-1.15 | | 0.20 | |
| FiO_2_ at time of ABG (Increment of 10%) | 0.91 | 0.81-1.03 | | 0.15 | |
| Maximum PaO_2_ (Increment of 1 fold) | 0.99 | 0.73-1.35 | | 0.97 | |
| APACHE at enrollment (Increment of 5) | 1.36 | 1.17-1.58 | | <0.001 | |
|  |  |  | |  | |
| ^1^: odds ratio for lower GCS. | | |  | |  |
